# Impact of Preemptive Analgesia on inflammatory responses and Rehabilitation after Primary Total Knee Arthroplasty: A Controlled Clinical Study

**DOI:** 10.1038/srep30354

**Published:** 2016-08-31

**Authors:** Xu Jianda, Qu Yuxing, Gao Yi, Zhao Hong, Peng Libo, Zhao Jianning

**Affiliations:** 1 Department of Orthopaedics, School of Medicine, Southern Medical University (Guangzhou), Jinling Hospital, 305 East Zhongshan Road, Nanjing, 210002, Jiangsu Province, China; 2 Department of Orthopaedics, Changzhou Traditional Chinese medical hospital, changzhou, 213003, China; 3 Department of Orthopaedics, Changzhou Traditional Chinese medical hospital, changzhou, 213003, China; 4 Department of Orthopaedics, School of Medicine, Southern Medical University (Guangzhou), Jinling Hospital, 305 East Zhongshan Road, Nanjing, 210002, Jiangsu Province, China.

## Abstract

The aim of this study was to investigate the effects of preemptive analgesia on the inflammatory response and rehabilitation in TKA. 75 patients with unilateral primary knee osteoarthritis were conducted in this prospective study. All patients were randomly divided into two groups (MMA with/without preemptive analgesia group). The following parameters were used to evaluate analgesic efficacy: knee flexion, pain at rest and walking, functional walking capacity (2 MWT and 6 MWT), WOMAC score, and hs-CRP level. Patients in MMA with preemptive analgesia group had lower hs-CRP level and less pain at rest and walking during the first week postoperatively (P < 0.05). The 2 MWT was significantly better in MMA with preemptive analgesia group (17.13 ± 3.82 VS 14.19 ± 3.56, P = 0.001). The 6 MWT scores and WOMAC scores increased significantly within Groups (P = 0.020, 0.000), but no difference between groups postoperatively (P > 0.05). Less cumulative consumption of morphine was found in MMA with preemptive analgesia group at 48 h (P = 0.017, 0.023), but no difference at total requirement (P = 0.113). Preemptive analgesia added to a multimodal analgesic regime improved analgesia, reduced inflammatory reaction and accelerated functional recovery at the first week postoperatively, but not improved long-term function.

The total knee arthroplasty (TKA) has been considered as a leading surgical treatment for advanced arthritis, but severe postoperative pain is still a major complaint of patients^1^. The postoperative pain directly results in a prolonged rehabilitation process and worse clinical outcome^2^,^3^. Ranawat *et al*. found that postoperative pain was related with arthrofibrosis which diminished range of motion^4^.

In latest decades, many substantial innovations improve perioperative analgesia, including continuous epidural analgesic technique, intravenous patient-controlled analgesia and peripheral nerve blockade. Although each method has been considered effectively in relieving postoperative pain, side effects can hardly be avoided. Multimodal approaches (MMA) provides a better choice with minimizing side effects^5^. Despite an extensive, multimodal analgesic approach, considerable acute pain is still appearance after TKA^6^. It’s believed that preemptive treatment can reduce the consumption of other analgesics, improve analgesic efficacy, and ameliorate immune function. As a selective cyclooxygenase (COX)-2 inhibitor, Celecoxib decreases the post-operative hyperalgesic state by inhibiting prostaglandin synthesis in the periphery and the spinal cord^7^. To achieve better postoperative analgesia for faster rehabilitation postoperatively, oral celecoxib preemptively was used.

Inflammatory reaction is a major component of surgical stress response^8^. Shen *et al*. found that C-reactive protein considerablly increased in patients with total knee arthroplasty^9^. C-reactive protein (CRP) is produced rapidly in response to inflammation, infection, tissue damage, and malignancy^10^. Sturmer *et al*. found that elevated levels of CRP was correlated with symptoms of pain, stiffness, and radiographic grading in OA^11^. To our knowledge, the effect of preemptive analgesia on the inflammatory response in TKA has not been studied.

The aim of this study was to investigate the effects of preemptive analgesia on the inflammatory response in patients with or without preemptive analgesia. In addition, we investigated whether preemptive analgesia leaded to low postoperative pain and better rehabilitation.

## Patients and Methods

The study protocol was approved by the Ethics Review Committee of Changzhou Traditional Chinese Medical Hospital, affiliated to Nanjing University of Traditional Chinese Medicine. Verbal and written informed consents were obtained before all study. The study was carried out in accordance with the approved guidelines.

From February 2013 to February 2014, seventy-five patients, ASA I–III, aged 54–76 (average, 68.3 ± 6.5) years, diagnosed with unilateral primary knee osteoarthritis(50 females and 25 males) were conducted in this prospective study. All enrolled patients were randomly allocated using a random-number table to receive MMA with/without preemptive analgesia. Exclusion criteria included inflammatory arthritis, alcohol or medical abuses, autoimmune disorder used steroid medication, and neurologic or psychiatric diseases potentially influencing pain perception.

All surgeries were done by one experienced orthopaedic surgeon with a specialist interest in arthroplasty. Prostheses were rotating platform for artificial knee joint (GEMINI MKII, Link Inc., Germany). The routine anaesthesia method were lumbar spinal anaesthesia (34 patients, 45.3%) or general Anaesthesia (41 patients, 54.7%). The tourniquet and wound drain were used in each case, and the drain was removed after 48 h postoperatively. Low Molecular Weight Heparin Sodium (Sanofi Winthrop Industrie, France, 0.6 ml:6000AxaIU) was used for thromboprophylaxis once daily post- operatively until discharge. The hospital stay was 14 days postoperatively. Mobilization started from the day of surgery with the same rehabilitation scheme by one physiotherapist. After satisfactory radiograph, patients would leave the bed ambulating with walking aids. While on the bed, they would sit on the edge of the bed dependent on their tolerance. On the following days, patients attended rehabilitation twice a day to perform exercises. According to the patient’s tolerance, the distance increased gradually.

Follow-up duration was defined as the date of operation to the date of last visit. Range of motion (ROM), pain at rest, pain at walking were measured at 8:30 a.m. during hospitalization. Hospital narcotic analgesic use, side-effects and complications were also recorded during hospitalization. After discharge, pain, ROM and functional results were evaluated at each follow-up. All parameters were measured before operation.

### Preemptive Analgesia and MMA

A preemptive oral analgesic regimen of celecoxib (200 mg, Pfizer, America; 400 mg, PO) was administered to each patient within 1 hour before operation without any other premedication^12^,^13^,^14^,^15^.

A standardized, multimodal analgesic regime with cocktail injection was administrated. The cocktail (combination of Ropivacaine 357.6 mg, morphine 5 mg, adrenaline 0.3 mg, ketorolac 30 mg) was administered pre- and post-cementation. The first injection was administered to posterior capsule, posteromedial structures and periarticular synovium. The second injection was administered to extensor mechanism, pes anserinus, anteromedial capsule, periosteum, iliotibial band and subcutaneous plane^16^.

As a primary analgesic, celecoxib (200 mg PO) was administered twice daily starting 12 hours postoperatively and continued throughout their hospital stay^17^. Subcutaneous morphine was used when the oral analgesics was not effective. All narcotic use was recorded. Post-operative nausea and vomiting (PONV) was treated with ondansetron.

### Analysis of blood samples

Systemic inflammation was measured by high-sensitivity C-reactive protein (hs-CRP) in venous blood before operation, daily at the first week, 2 weeks, 1, 3, 6 months after surgery. Venous blood sample was taken from each patient at 7 a.m.

Hs-CRP was tested by Simens BN II analyser (Simens Healthcare Diagnostics Products GmbH, Marburg, Germany) according to the manufacturer’s instructions. The normal range for Hs-CRP values: Low risk ≤1 mg/L. Normal = 1–3 mg/L. High risk ≥3 mg/L.

### Study clinical parameters

#### Knee Flexion

Knee flexion was measured in supine lying using a goniometer^18^.

#### Pain

The quality of postoperative pain relief was measured by morphine consumption and the visual analogue scale. Patients were asked to mark their level of pain on a 10 cm visual analogue scale^19^. Pain at rest was evaluated after 30 min rest. Pain on walking or knee maximal flexion was also recorded.

### Functional walking capacity

The 2 min walk test (2 MWT) and 6 min walk test (6 MWT) were used to evaluate functional walking capacity^20^. The distance covered was recorded in metres. Baseline-predicted 2 MWT and 6 MWT distance was calculated using gender-specific reference equations for the 6 MWT^21^.

### Assessment of knee function

The Western Ontario and McMaster Universities Osteoarthritis Index (WOMAC) was administered to assess the knee function^22^.

### Statistical analysis

The normality of distribution for continuous numeric variables was assessed by Kolmogorov–Smirnov test. According to normally distributed or not, the variables are presented as means with SD, and otherwise as medians with inter-quartile ranges (95% confidence intervals, 95% CI). Student’s t-test for normally distributed continuous variables, while others using Pearson’s χ^2^ test or Wilcoxon’s rank sum test.

Continuous data with repeated measures were analysed using repeated-measures regressions for general linear models. Bivariate analysis was used to analysis the relation of different studied variables.

The SPSS statistical package (Version 17.0, SPSS Inc., Chicago, IL, USA) was used for statistical analyses. P < 0.05 was considered as a positive significance.

## Results

Seventy-five patients were assessed for eligibility in this study and randomly divided into two groups (MMA with/without preemptive analgesia group). Baseline pre- and perioperative was similar in both groups (Table 1).

### hs-CRP and Pain

**Figure 1:**
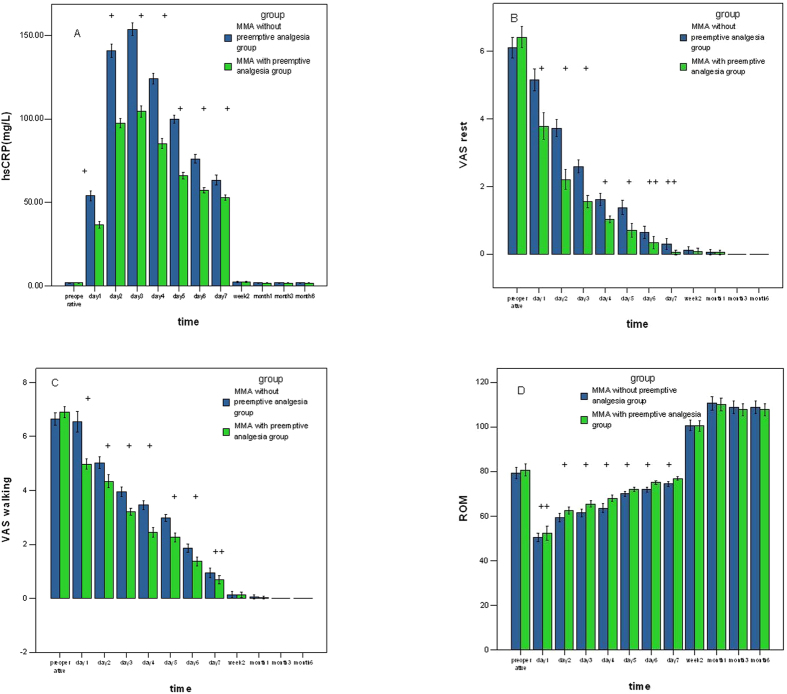
(**A**) Hs-CRP level after arthroplasty. Tests with repeated measurement regression. (**B,C**) Pain at rest and walking after arthroplasty. Tests with repeated measurement regression. (**D**) Range of motion (ROM) after arthroplasty. Tests with repeated measurement regression. (^+^P < 0.01; ^++^P,0.05. Blue: MMA without preemptive analgesia group; Green: MMA with preemptive analgesia group).

Patients in MMA with preemptive analgesia group had lower hs-CRP level during the first week postoperatively (P < 0.05, Fig. 1A). The CRP levels in both groups increased rapidly and peaked 3 days postoperatively at maximum levels(104.46 ± 10.67 Vs 153.63 ± 11.11).

No difference was found after 7 days postoperatively. The CRP then fell to preoperative values gradually and eventually returned to normal by 2 weeks postoperatively.

The hs-CRP level was positively correlated with pain at rest and walking (correlation coefficient 0.398/0.377, P = 0.000, 0.000), but negatively correlated with ROM (correlation coefficient −0.741, P = 0.000).

### Pain and rescue analgesic requirement

Less pain at rest and walking were found in MMA with preemptive analgesia group (P < 0.05), but no difference after the first week. No significance was found about severe pain at rest or during walking on the further follow-up (Fig. 1B,C).

On the follow-up of three months, one patient in MMA with preemptive analgesia group and two patients in MMA without preemptive analgesia group reported moderate pain during walking and recovered by celecoxib.

Less cumulative consumption of morphine was found in the MMA with preemptive analgesia group from 0 to 48 h (P = 0.017, 0.023, Table 2), but no total morphine requirement difference during the remaining study period (P = 0.113, Table 3).

### Functional results

#### Early postoperative functional outcome measures

All patients leaved the bed ambulating with weight-bearing with walking aids on days 2 or 3.

The 2 MWT was significantly better in MMA with preemptive analgesia group (17.13 ± 3.82 VS 14.19 ± 3.56, P = 0.001), but no differences on day 7 and week 2 (P = 0.148, 0.108, Table 4). ROM) after arthroplasty has the same results (Fig. 1D).

#### Late postoperative functional outcome

The preoperative 6 MWT was similar in both groups (P = 0.392). When repeated 1, 3, 6 months postoperatively, the 6 MWT scores increased significantly within groups (P = 0.020), but no difference was found in both groups (P = 0.153, 0.735, 0.973 ).

Similarly, the changes in WOMAC scores were statistically significant (P = 0.000), with no difference between groups (P = 0.914, 0.111, 0.992, Table 3).

#### Postoperative Complications

There was no obvious side-effects or complications related to drug during hospitalization or on further follow-up. In present study, no superficial or deep infections occurred (Table 4). None bleeding complication was found in this current study.

The incidence of PONV and No. of patients requiring ondansetron were overall low. Compared with MMA without preemptive analgesia group, no significant difference was found about the number of patients with nausea, vomiting and requiring ondansetron (P > 0.05, Table 4).

## Discussion

Surgical trauma usually induces an inflammatory state with the release of pro- and anti-inflammatory proteins. Surgeons recognized that suppression of the inflammatory response could decrease the complications, due to less inflammatory mediators in nociceptive processing^23^,^24^. Preemptive analgesia is initiated to prevent the peripheral and central nociception before the noxious stimulus arises. To overcome inflammatory responses, different drugs are being tried by the technique. These trials cover NSAIDs, opioids, NMDA receptor antagonists and systemic antiepileptics^25^,^26^,^27^,^28^. Stephens *et al*. had found that NSAIDs was important in extending the analgesia of multimodal analgesia, and improved clinical outcomes after surgery^29^. Considering the aim of attenuating the peripheral and central nociception and side effects, we preferred dedicated COX-2 inhibitor celecoxib as preemptive analgesia oral analgesic regimen. Digestive complications, homeostasis, and negative effects on bone repairing will be improved by taking selective inhibitors of Cox-2 in comparison to ordinary NSAID^30^. Preemptive analgesia oral analgesic regimen of celecoxib could effectively attenuate the establishment of hyperalgesia by suppressing afferent and efferent fibers by attenuating inflammatory response.

In present study, we did see a slash reduction in hs-CRP level and pain score in the MMA with preemptive analgesia group. The level of hs-CRP increased in both groups, with a peak at the third day post-operatively and gradually normalized. In both groups, hs-CRP level rised faster compared with latter degression.

The pain was significantly reduced in the first week postoperatively in MMA with preemptive analgesia group. No difference was found after 1week postoperatively. Despite we prolonged the assessment of pain upon rest and walking, no prolonged analgesic effect was observed. Lunn TH, *et al*. have demonstrated that a single preoperative high dose of MP (125 mg i.v.) could reduce the overall pain for the first 48 h after TKA, and improve analgesia and immediate recovery postoperatively^31^. De Oliveira GS *et al*. concluded that a certain amount of glucocorticoid was necessary for reducing opioid consumption and postoperative pain^32^. Hall GM *et al*. studied the relationship between inflammatory responses and functional recovery in hip arthroplasty, and concluded that the inflammatory response was related with immediate functional recovery. And attenuation of the inflammatory meant less pain and better functional recovery^33^.

Celecoxib could effectively attenuate the hyperalgesia of afferent and efferent fibers by attenuating inflammatory response. Previous study had concluded that local anesthetics could modulate the inflammatory response in mice systemically or locally^34^. Sinclair *et al*. found that amide local anesthetics could suppress markedly the metabolic activation and secretory function of leukocytes in a dose-dependent manner^35^. This may explain the difference of hs-CRP levels in MMA with/without preemptive analgesia group was mediated by preemptive analgesia oral analgesic regime.

The nerve fibers around surgical area of the knee covers: the femoral nerve (anterior), the obturator nerve (medial), the lateral femoral cutaneous nerve (lateral) and the sciatic nerve (posterior). Periarticular infiltration of local anaesthetics may provide good analgesia locally and improve walking capacity^36^. Therefore, we administered local injection to all areas of the knee. And the pain intensity in the present study showed adequately nerve blocked.

Because of less use of opioids in both groups, PONV was not a serious clinical problem^37^, and no significant difference was found.

Our study did not supply a more detailed explanation for the preemptive analgesic benefit. However, suppressing the system inflammatory response (hs-CRP) means less inflammatory mediators in nociceptive processing^23^.

Our study had several strengths: this was a prospective controlled study. All patients were screened and followed at one single centre and received standardized pre-, peri-, and postoperative regime. The detailed assessment of outcomes was collected by one single data collector, resulting in few missing data.

However, there were also limitations to our study, including a relatively small sample size. Nevertheless the small number of patients illustrated the importance of preemptive analgesia on inflammatory response and postoperative functional outcome.

In conclusion, preemptive analgesia oral analgesic regimen added to a multimodal analgesic regime improved analgesia, reduced inflammatory reaction and accelerated functional recovery atthe first postoperative week, but not improved long-term function. Therefore, these outcomes are important to determine optimal pain management after TKA.

## Additional Information

**How to cite this article**: Jianda, X. *et al*. Impact of Preemptive Analgesia on inflammatory responses and Rehabilitation after Primary Total Knee Arthroplasty: A Controlled Clinical Study. *Sci. Rep.* **6**, 30354; doi: 10.1038/srep30354 (2016).

## Figures and Tables

**Table 1:** Baseline patient characteristics and pre- and perioperative characteristics.

Variable	MMA with preemptive analgesia group	MMA without preemptive analgesia group	*P*-value
Patient characteristics			
Age (yr)	67.8 ± 7.2	68.8 ± 6.5	0.106
Gender (male/female)	12/26	13/24	0.467
BMI (kg m-2)	27.2 ± 2.7	26.8 ± 3. 1	0.08
ASA (I/II/III)	2/26/10	2/29/6	0.370
Co-morbidities			
None	11	9	0.886
Hypertension	15	17	
Diabetes	6	8	
Coronary artery disease	3	2	
others	2	1	
Side of surgery (R/L)	10/28	12/25	0.372
Preoperative data			
Pain at rest	6.11 ± 0.91	6.22 ± 0.95	0.148
Pain at walking	6.65 ± 0.68	6.89 ± 0.61	0.101
Hs-CRP (μg/ml)	1.99 ± 0.55	1.94 ± 0.52	0.644
Perioperative data			
Duration of surgery (min)	58.5 ± 8.6	56.1 ± 8.4	0.686
Bleeding intraoperatively (ml)	423.5 ± 95.8	414.5 ± 98.4	0.207
Duration of femoral tourniquet (min)	24.6 ± 6.4	23.9 ± 6.1	0.312

**Table 2:** Cumulative consumption of morphine after total knee arthroplasty.

Time	MMA with preemptive analgesia group	MMA without preemptive analgesia group	*P*-value between groups
0–24 h	13.25 ± 7.67	18.05 ± 8.57	0.017
24–48	19.08 ± 7.24	23.05 ± 9.24	0.023
total	43.28 ± 14.65	45.03 ± 15.42	0.113

**Table 3:** Postoperative functional outcome after total knee arthroplasty.

Variables	MMA with preemptive analgesia group	MMA without preemptive analgesia group	*P*-value between groups
2 min walk test (m) preoperative			
3 d postoperatively	17.13 ± 3.82	14.19 ± 3.56	0.003
1 w postoperatively	34.08 ± 5.68	32.05 ± 6.31	0.149
2 w postoperatively	71.05 ± 8.65	67.54 ± 9.99	0.108
P-value within groups	0.000		
6 min walk test (m)			
preoperative	164.53 ± 27.99	163.32 ± 31.03	0.392
1 m postoperatively	205.29 ± 48.11	195.57 ± 44.39	0.153
3 m postoperatively	250.29 ± 46.92	262.16 ± 47.82	0.735
6 m postoperatively	247.92 ± 35.70	266.05 ± 39.58	0.973
P-value within groups	0.020		
WOMAC			
preoperative	50.74 ± 7.03	51.87 ± 7.47	0.503
1 m postoperatively	24.03 ± 7.25	23.84 ± 7.76	0.914
3 m postoperatively	11.84 ± 4.59	10.19 ± 4.27	0.111
6 m postoperatively	6.90 ± 1.25	6.89 ± 1.22	0.992
P-value within groups	0.000		

**Table 4:** Nausea, vomiting, and consumption of ondansetron during Perioperative period.

Variable	MMA with preemptive analgesia group	MMA without preemptive analgesia group	*P*-value
No. of patients with nausea			
6 h	6	6	0.603
24 h	5	9	0.203
2 w	0	0	—
No. of patients with episode (s) of vomiting			
6 h	3	4	0.515
24 h	2	3	0.513
2 w	0	0	—
No. of patients requiring ondansetron			
6 h	1	2	0.572
24 h	1	1	0.747
2 w	0	0	—
